# Knowledge graph revision in the context of unknown knowledge

**DOI:** 10.1371/journal.pone.0302490

**Published:** 2024-07-05

**Authors:** Shuangmei Wang, Fengjie Sun

**Affiliations:** 1 Jilin University, Changchun, China; 2 Hainan University, Haikou, China; Government College University Lahore, PAKISTAN

## Abstract

The role of knowledge graph encompasses the representation, organization, retrieval, reasoning, and application of knowledge, providing a rich and robust cognitive foundation for artificial intelligence systems and applications. When we learn new things, find out that some old information was wrong, see changes and progress happening, and adopt new technology standards, we need to update knowledge graphs. However, in some environments, the initial knowledge cannot be known. For example, we cannot have access to the full code of a software, even if we purchased it. In such circumstances, is there a way to update a knowledge graph without prior knowledge? In this paper, We are investigating whether there is a method for this situation within the framework of Dalal revision operators. We first proved that finding the optimal solution in this environment is a strongly NP-complete problem. For this purpose, we proposed two algorithms: Flaccid_search and Tight_search, which have different conditions, and we have proved that both algorithms can find the desired results.

## 1 Introduction

A knowledge graph is a graphical structure used to organize and represent knowledge. As artificial intelligence (AI) advances, the importance of knowledge graph in the field of AI is becoming increasingly prominent [1]. It is a network composed of entities (such as people, places, and things) and how they’re connected. It’s meant to help computer systems understand and figure out information better. The goal of a knowledge graph is to simulate the human knowledge system, enable computers to process information in a more intelligent way. It serves not only as a tool for storing and retrieving knowledge but also supports applications such as semantic search, automatic question answering, and recommendation systems. By integrating knowledge from different domains into a unified structure, knowledge graphs contribute to building more comprehensive and accurate data models, providing robust support for artificial intelligence and big data analytics.

As time progresses, information in the real world continually evolves and changes, leading to the potential obsolescence or inclusion of inaccurate data within the knowledge graph. To ensure the knowledge graph reflects the most current real-world situations, regular revisions and updates are essential. Revision may involve adding new entities and relationships, deleting outdated or incorrect information, and adjusting the weights and associations of existing data. Through such revision processes, the knowledge graph can better serve user needs by providing an accurate and reliable knowledge foundation, supporting various application domains such as search, recommendation systems, and intelligent decision-making.

The propositional logic formulas can be used to represent relationships and entities in knowledge graph [2]. In propositional logic, proposition symbols are used to represent different propositions or statements, and logical operators are used to express relationships between them. For instance, Let’s we have the following knowledge graph:

- There is a relationship *R* between entity *A* and entity *B*, then it can be represented as *R* ∧ (*A* → *B*).
- There is a relationship *S* between entity *B* and entity *C*, then it can be represented as *S* ∧ (*B* → *C*).

In this paper, we are concerned with the problem that the knowledge graph can be revisied by introduction new entities and relationship in the environment that the knowledge is unknown. Suppose that we have *n* knowledge graphs, each with a set of current knowledge graph *K*_*i*_ and an epistemic goal *ψ*_*i*_, in which $K_{i}=K_{i}^{\prime }\cup K_{i}^{\prime \prime }$, $K_{i}^{\prime }$ where $K_{i}^{\prime }$ is the knowledge graph *i* that has been konwn, and $K_{i}^{\prime \prime }$ is the knowledge graph *i* that has not been konwn. We are interested in finding a single formula *ϕ* such that each knowledge graph *K*_*i*_ can achieve the corresponding goal *ψ*_*i*_ after receive the formula *ϕ*. In this paper, we will research the following problem:

1. We are conducting research on the Knowledge Graph Revision Problem with Limited Knowledge (LKG) problem and establishing the LKG problem model.
2. We are researching the complexity of the LKG problem.
3. To address this problem, we have proposed two approximate search algorithms: Flaccid_search and Tight_search, and analyzed the performance and characteristics of these two algorithms.

## 2 Related work

### 2.1 Dynamic epistemic logic

The researchers in [3] have proposed that Dynamic Epistemic Logic (DEL) refers to a class of modal logics for reasoning about actions and belief. The researchers in [4] have proposed that the announcement problem is addressed in arbitrary public announcement logic (APAL). The researchers in [5] have proposed that the model checking problem of APAL is in PSPACE. The researchers in [6] have proposed that the model checking of APAL in its succinct form is NEXPTIME complete. The researchers in [7] have proposed that the relevance of such symbolic models and show that the symbolic model checking problem against PAPL is a EXPTIME-complete as soon as announcement protocols allow for either arbitrary announcements or iteration of public announcements. The researchers in [8] have proposed that the satisfiability problem of APAL is undecidable, but gets decidable when announcement sare propositional [9].

The researchers in [10] have proposed that one highly influential approach to belief revision is the AGM approach, in which revision is captured by an operator thatsatisfies a particular set of rationality postulates. The researchers in [11] have proposed that every AGM revision operator * has the property that, for any belief set *K*, there is an underlying total pre-order ≺_*K*_ over interpretations of *P* such that $\left|K*\phi \right|=min_{{≺}_{K}}\left(\phi \right)$. The researchers in [12] have proposed the Dalal operator denoted by *_d_, which underlying ordering is defined by the Hamming distance between interpretations. The researchers in [13] have proposed that with some revision operators, agents can be manipulated to believe a target formula *ϕ* exclusively through indirect statements or evidence. [14] considered a related problem called inverse revision (IR): when is *K*_*i*_ *_i_*ϕ* = *ψ*_*i*_ for all agents ipossible, where and *ϕ* any AGM-style revision operators *_i_ can be chosen; the set of all such *ϕ* is called the frame, which is complete if any *ϕ* and *ϕ*′ are logically equivalent. Checking whether a formula *ϕ* is in the frame is co-NP-complete, and deciding frame-completeness is $\Pi_{2}^{n}$-complete. The researchers in [15] have proposed that the not necessarily truthful public announcements in the setting of AGM belief revision and proved that announcement finding in this setting is not only decidable, but that it is simpler than the corresponding problem in the most simplified modal logics. The researchers in [16] have studied the problem of the existence of such an announcement in the context of model-preference definable revision operators.

### 2.2 Knowledge Graph (KG)

Knowledge representation has a long history of development in the fields of logic and AI. As artificial intelligence advances, the importance of knowledge graph in the field of AI is becoming increasingly prominent [1]. Firstly, integrating deep learning with knowledge representation and reasoning can enable systems to better understand and utilize vast amounts of knowledge [17]. For example, using deep learning to extract knowledge from large text data and representing it in a computationally processable form to support construction of Static KGs and decision-making [18]. Many researchers utilize neural network architectures, such as CNNs. The researchers in [19] employ convolutional layers and fully-connected layers to train knowledge graph embedding vectors. The researchers in [20] utilize standard convolutions, atrous convolutions, and residual networks in its neural network architecture. The researchers in [2] have proposed three types of negatives: in-batch negatives, pre-batch negatives, and self-negatives which act as a simple form of hard negatives. Combined with InfoNCE loss, the proposed model SimKGC can substantially outperform embedding-based methods on several benchmark datasets. The researchers in [21] have proposed that new capabilities to the model, enabling it to process texts in various domains such as geographical areas, transportation, organizations, literary works, biology, natural sciences, astronomical objects, and architecture. Secondly, the fusion of different forms of representation is key to enhancing the capability of knowledge representation and reasoning. Logical representation is suitable for reasoning and decision-making, probabilistic graphical models are suitable for uncertainty reasoning, and graph structures are suitable for representing complex relationships, among others. Integrating different forms of representation can provide a more comprehensive and flexible capability for knowledge representation and reasoning [22]. For example, temporal KGs including time information in their data which can constantly gather and update information to maintain the currency of knowledge [23]. Thirdly, incorporating background knowledge can enhance the accuracy and robustness of systems [24]. For example, using ontology technology to construct domain ontologies and combining them with inference mechanisms to support deeper and more precise reasoning [25]. Fourthly, as the scalability of reasoning improves, the computational complexity of reasoning also increases [15]. Therefore, researching more efficient reasoning algorithms and technologies to improve the speed and scalability of reasoning is crucial [26]. For example, using parallel computing and distributed computing technologies can accelerate the reasoning process [27]. Finally, incorporating human knowledge and judgment can construct AI systems with more human-like intelligence [28]. By collaborating with domain experts and integrating their experience and intuition into knowledge representation and reasoning, more accurate and trustworthy reasoning results can be provided [29]. Therefore, combining deep learning, integrating different forms of representation, incorporating background knowledge, improving reasoning efficiency and scalability, and incorporating human knowledge and judgment can drive greater breakthroughs and progress in knowledge representation and reasoning in AI systems [30]. However, in many fields, acquiring training data for deep learning faces challenges. Particularly in cases involving personal privacy, trade secrets, or requiring extensive annotation, data acquisition is subject to strict legal and ethical requirements. Fields such as healthcare, military and intelligence, finance, and natural disaster prediction fall into this category of hard-to-obtain data. In these fields, protecting the privacy and security of data is crucial, requiring additional measures to ensure the lawful use of data [31].

## 3 Preliminaries

A Knowledge Graph is a formalized framework for representing knowledge, with its core concept being the modeling of real-world information in a graphical structure. In a formal representation, a knowledge graph can be defined as a triple set *G* = *E*, *R*, *T*, where:

- *E* represents the set of entities, denoting individuals, objects, or concepts in the real world.
- *R* represents the set of relations, describing the semantic associations between entities.
- *T* represents the set of triples, with each triple (*t*, *r*, *t*′) representing the connection between two entities through relation *r*.

The Knowledge Revision Problem is that there have *n* agents and *n* AGM knowledge revision operators *_i_ input; we are looking for the existence of a consistent formula *ϕ* such that [15]

$$\begin{array}{c} \begin{array}{c} K_{1}{*}_{1}\phi =\psi_{1}\\ K_{2}{*}_{2}\phi =\psi_{2}\\ \vdots \\ K_{n}{*}_{n}\phi =\psi_{n} \end{array} \end{array}$$ (1)

where *K*_*i*_ and *ψ*_*i*_ represent the knowledge graph set and the goal of the agent *i*, respectively. Obviously, if ⋀_*i*_*ψ*_*i*_ is consistent, then we can just revise by this [15]. In some cases, however, not all the knowledge graph set of agents are completely known, then $K_{i}=K_{i}^{\prime }\cup K_{i}^{\prime \prime }$, $K_{i}^{\prime }$ where $K_{i}^{\prime }$ is the the knowledge graph set of agent *i* that has been konwn, and $K_{i}^{\prime \prime }$ is the the knowledge graph set of agent *i* that has not been konwn.

**Example 1** *Consider the UAV controller example over the vocabulary patrol; exitinguishing. We think of this vocabulary as defining a state machine, where each interpretation represents a state; the UAV can have actions that are triggered by transitions to given states. This is a standard control mechanism for simple agents in a video game setting, and it can function as a control mechanism for our simple UAVs as well. In our example, the patrol variable is true when the UAV should be patrolling their area and the exitinguishing variable is true when the UAV receive an alarm due to fire*.

*Suppose we have two UAVs U*_1_ *and U*_2_ *with initial knowledge states defined as follows*:

$$\begin{array}{c} \begin{array}{cc} & Bel\left(U_{1}\right)=\left\{\neg patrol\vee exitinguishing\right\}\\ & Bel\left(U_{2}\right)=\left\{\neg patrol\vee \neg exitinguishing\right\} \end{array} \end{array}$$

*The controller receive an alarm due to fire. Is there a formula that can be broadcast to immediately get U*_1_ *to exitinguishing the fire while U*_2_ *keep patrolling? In other words, is there a formula ϕ such that*:

$$\begin{array}{c} \begin{array}{cc} & \{\neg patrol\vee exitinguishing\}*\phi ⊢exitinguishing\\ & \{\neg patrol\vee exitinguishing\}*\phi ⊢\neg exitinguishing\vee patrol \end{array} \end{array}$$

*The answer is yes; we can set ϕ* = *patrol*.

The preceding example is framed in the context of the UAV controller, but it also demonstrates an important case for propositional announcement. In particular, it shows that there are cases where the goals are inconsistent, yet a solution is possible. The researchers in [15] have proposed the EXIST_ANN Algorithm to solve this problem. The process of the EXIST_ANN is described in the Algorithm 1.

**Algorithm 1** EXIST_ANN(*K*_1_, *K*_2_⋯*K*_*n*_; *ψ*_1_, *ψ*_2_⋯*ψ*_*n*_)

**Require**: *K*_1_, *K*_2_⋯*K*_*n*_; *ψ*_1_, *ψ*_2_⋯*ψ*_*n*_

**Ensure**: *v*_1_, *v*_2_⋯*v*_*n*_

1: Let *m* be the size of the underlying input vocabulary of *K*_1_, *K*_2_⋯*K*_*n*_; *ψ*_1_, *ψ*_2_⋯*ψ*_*n*_.

2: Guess *d*_1_, *d*_2_⋯*d*_*n*_ ∈ {0, 1⋯*m*}.

3: Guess *v*_1_, *v*_2_⋯*v*_*n*_.

4: **for all** *i*, *j* ∈ {1, 2⋯*n*} **do**

5:  **if** *d*(*k*_*j*_, *v*_*i*_) < *d*_*j*_ **then**

6:   Reject.

7:  **end if**

8:  **if** *d*(*k*_*j*_, *v*_*i*_) > *d*_*j*_ **then**

9:   Reject.

10:  **end if**

11:  **if** *d*(*k*_*j*_, *v*_*i*_) = *d*_*j*_ and *v*_*i*_ ⊭ *ψ*_*j*_ **then**

12:   Reject.

13:  **end if**

14: **end for**

15: Accept.

The EXIST_ANN Algorithm is effective for solving the Propositional Announcement Problem, however, in some environments, the initial knowledge set of agents cannot be known. In this environment, the EXIST_ANN Algorithm cannnot obtain an effective solution. Next, we will explain in detail with the Example 2.

**Example 2** *Assume the vocabulary* {*a*, *b*, *c*}, *the knowledge et of the agent A*_1_ *is K*_1_ *and the knowledge set of the agent A*_2_ *is K*_2_ *in which* $K_{1}=K_{1}^{\prime }\cup K_{1}^{\prime \prime }$ *and* $K_{2}=K_{2}^{\prime }\cup K_{2}^{\prime \prime }$, *where for* $i\in \left\{1,2\right\},K_{i}^{\prime }$ *is the the knowledge set of agent A*_*i*_ *that has been konwn, and* $K_{i}^{\prime \prime }$ *is the the knowledge set of agent A*_*i*_ *that has not been konwn. let* $K_{1}^{\prime }=\left\{a;\neg b\to c;b\to \neg c\right\},K_{1}^{\prime \prime }=\left\{a\to b\right\}$ *and* $K_{2}^{\prime }=\left\{\neg c\right\},K_{2}^{\prime \prime }=\left\{\neg b\right\}$, *ψ*_1_ = {¬*a*; *c*}, *ψ*_2_ = {¬*b*; *c*}.

*Through the EXIST_ANN Algorithm, we can conclude that d*_1_ = 1, *d*_2_ = 1, *and there exists a solution that ϕ* = ¬*a* ∧ ¬*b* ∧ *c*. *This is a wrong solution because the minimum hamming distance d*_*min*_(*K*_1_, *ϕ*) = 2. *Therefore, the EXIST_ANN Algorithm cannot deal with the problem in the environment that the initial knowledge set of agents are unknown*.

## 4 The knowledge graph revision problem with limited knowledge

### 4.1 Problem definition

In this section, we restrict the problem slightly by requiring that all agents have the same revision operator. That is given *n* agents and an AGM revision operator *, we are looking for if there exists a consistent formula *ϕ* such that

$$\begin{array}{c} \begin{array}{c} K_{1}*\phi =\psi_{1}\\ K_{2}*\phi =\psi_{2}\\ \vdots \\ K_{n}*\phi =\psi_{n} \end{array} \end{array}$$ (2)

where *K*_*i*_ and *ψ*_*i*_ represent the knowledge set and the goal of the agent *i*, respectively. In principle, * could be any shared revision operator. In some cases, however, not all the knowledge set of agents are completely known, thus $K_{i}=K_{i}^{\prime }\cup K_{i}^{\prime \prime }$, where $K_{i}^{\prime }$ is the the knowledge set of agent *i* that has been konwn, and $K_{i}^{\prime \prime }$ is the the knowledge set of agent *i* that has not been konwn.

In practice, we will often be interested in the complexity of finding announcements; but we must first consider the corresponding decision problem. The formal definition is as follows:

**Definition 4.1** *The Knowledge Graph Revision Problem with Limited Knowledge*

*Input*:

 *An integer n*

 *A list of the initial knowledge* $K_{1}^{\prime },K_{2}^{\prime }\cdots K_{n}^{\prime }$.

 *A list of ψ*_1_, *ψ*_2_⋯*ψ*_*n*_ *formulas (goals)*.

*Ouput*:

*Yes, if there exists ϕ satisfying* Eq 2

*No, otherwise*.

We refer to this problem as *LKG*(*), which emphasizes that it depends on some given operator * on knowledge sets. We normally assume that * is an AGM revision operator, but this need not be the case in general.

Next, we still need to further explore the complexity of the *LKG*(*_d_) problem. This problem can be seen as two stages: the stage one is the estimation of *v*_*i*_; the stage two is to check the the minimal Hamming distance between *v*_*i*_ and $K_{i}^{\prime }$.

### 4.2 Problem solution

For the moment, we are interested in analyzing a simple case to obtain the most efficient algorithm possible. Hence, we consider Dalal’s well-known revision operator based on Hamming distance [12]. In this section, we let *_d_ denote Dalal’s revision operator.

We present the Flaccid_search Algorithm and the Tight_search Algorithm to solve the *LKG*(*_d_) problem. In the algorithm, we use non-determistic choice to select the minimum distance *d*_*i*_ between $K_{i}^{\prime }$ and *ψ*_*i*_. Also note that *d*(*w*, *v*) denotes the Hamming distance between the set of models of interpretation *w* and interpretation *v*. The process of the Flaccid_search algorithm has been shown in Algorithm 2.

**Algorithm 2** Flaccid_search $\left(K_{1}^{\prime },K_{2}^{\prime }\cdots K_{n}^{\prime };\psi_{1},\psi_{2}\cdots \psi_{n}\right)$

**Require**: $K_{1}^{\prime },K_{2}^{\prime }\cdots K_{n}^{\prime };\psi_{1},\psi_{2}\cdots \psi_{n}$; ϕ=⌀

**Ensure**: *v*_1_, *v*_2_⋯*v*_*n*_

1: Let *m* be the size of the underlying input vocabulary of $K_{1}^{\prime },K_{2}^{\prime }\cdots K_{n}^{\prime };\psi_{1},\psi_{2}\cdots \psi_{n}$.

2: Estimate *d*_1_, *d*_2_⋯*d*_*n*_ ∈ {0, 1⋯*m*}.

3: Estimate *v*_1_, *v*_2_⋯*v*_*n*_.

4: **for all** *i* ∈ {1, 2⋯*n*} **do**

5:  **if** $\exists w⊧K_{i}^{\prime },d\left(w,v_{i}\right)=d_{i}$ **then**

6:   **if** *v*_*i*_ ⊨ *ψ*_*j*_ **then**

7:    *ϕ* = ⋀_*i*_{*v*_*i*_}

8:   **else** Reject.

9:   **end if**

10:  **else** Reject.

11:  **end if**

12: **end for**

The Line 1 of Algorithm 2 is estimate the value of *m* according to $K_{1}^{\prime },K_{2}^{\prime }\cdots K_{n}^{\prime }$ and *ψ*_1_, *ψ*_2_⋯*ψ*_*n*_. (i.e. *m* is the number of vocabulary of $K_{2}^{\prime }\cdots K_{n}^{\prime }$ and *ψ*_1_, *ψ*_2_⋯*ψ*_*n*_), The Line 2 of Algorithm 2 is estimate the value of *d*_*i*_, the value of which is {0, 1⋯*m*}. The Line 3 of Algorithm 2 is estimate the value of *v*_*i*_. The Line 4 to Line 12 of Algorithm 2 is the process of judging whether *v*_*i*_ is acceptable, if it is acceptable, add *v*_*i*_ to *ϕ*, and at the initial state *ϕ* is an empty set. However, although the judgment condition of the Algorithm 2 is relatively loose, there will still be situations where the Algorithm 2 has no solution. Next, we will explain in detail through the Proposition 4.1.

**Proposition 4.1** *There exist instances that the Algorithm 2 has no solution*.

**Proof**. Assume the vocabulary {*a*}, let *K*_1_ = {*a*}, *K*_2_ = {¬*a*}, *ψ*_1_ = ¬*a*, *ψ*_2_ = *a*, *d*_1_ = 0, *d*_2_ = 0, then we can conclude immediately that there is no solution of Algorithm 2.

Next, We need to prove that if there exists *ϕ* satisfying the *LKG*(*_d_), then the Flaccid_search $\left(K_{1}^{\prime },K_{2}^{\prime }\cdots K_{n}^{\prime };\psi_{1},\psi_{2}\cdots \psi_{n}\right)$ can produce the accepted result. In the proof, we write *d*(*x*_*i*_, *v*_*i*_) for the minimal Hamming distance from a model of *v*_*i*_ to a model of *x*_*i*_.

**Theorem 4.1** *Let* $\bar{K}=K_{1},K_{2}\cdots K_{n}$ *and let* $\bar{\psi }=\psi_{1},\psi_{2}\cdots \psi_{n}$ *be sequences of formulas. Then Flaccid_search* $\left(\bar{K},\bar{\psi }\right)$ *accepts if there exists ϕ such that K*_*i*_ *_d_ *ϕ* ⊨ *ψ*_*i*_ *for each i*.

**Proof**. Because there exists *ϕ* such that *K*_*i*_ *_d_ *ϕ* ⊨ *ψ*_*i*_ for each *i*, according the Eq 2, for all *i* ∈ {1, 2⋯*n*}, there exists *v*_*i*_ ⊨ *K*_*i*_ and *d*(*x*_*i*_, *v*_*i*_) = *d*_*i*_. Thus the Algorithm 2 will be accepted.

**Algorithm 3** Tight_search $\left(K_{1}^{\prime },K_{2}^{\prime }\cdots K_{n}^{\prime };\psi_{1},\psi_{2}\cdots \psi_{n}\right)$

**Require**: $K_{1}^{\prime },K_{2}^{\prime }\cdots K_{n}^{\prime };\psi_{1},\psi_{2}\cdots \psi_{n}$; ϕ=⌀

**Ensure**: *v*_1_, *v*_2_⋯*v*_*n*_

1: Let *m* be the size of the underlying input vocabulary of $K_{1}^{\prime },K_{2}^{\prime }\cdots K_{n}^{\prime };\psi_{1},\psi_{2}\cdots \psi_{n}$.

2: Estimate *d*_1_, *d*_2_⋯*d*_*n*_ ∈ {0, 1⋯*m*}.

3: Estimate *v*_1_, *v*_2_⋯*v*_*n*_.

4: **for all** *i* ∈ {1, 2⋯*n*} **do**

5:  **if** $\forall w⊧K_{i}^{\prime },d\left(w,v_{i}\right)=d_{i}$ **then**

6:   **if** *v*_*i*_ ⊨ *ψ*_*i*_ **then**

7:    *ϕ* = ⋀_*i*_{*v*_*i*_}

8:   **else** Reject.

9:   **end if**

10:  **else** Reject.

11:  **end if**

12: **end for**

The process of the Tight_search Algorithm is similar to the the Flaccid_search Algorithm, except the more stringent judgment conditions. The process of the Flaccid_search algorithm has been shown in Algorithm 3. However, although the judgment condition of the Algorithm is strict, there will still be situations where the Algorithm 3 has a solution. Next, we will explain in detail through the Proposition 4.2.

**Proposition 4.2** *There exist instances that the Algorithm 3 has a solution*.

**Proof**. Assume the vocabulary {*a*, *b*, *c*}, let *K*_1_ = {¬*a* → *b*; *a* → ¬*b*}, *K*_2_ = {¬*b*; ¬*c*}, *ψ*_1_ = {*a*; *b*}, *ψ*_2_ = {*b*; *c*}, *d*_1_ = 0, *d*_2_ = 0, then we can conclude immediately that there exists a solution of Algorithm 3 that *ϕ* = *a* ∧ *b* ∧ *c*.

Next, we need to prove that if the Tight_search(*K*_1_, *K*_2_⋯*K*_*n*_; *ψ*_1_, *ψ*_2_⋯*ψ*_*n*_) produce the accepted result *ϕ*, then *ϕ* can satisfy the *LKG*(*_d_). In the proof, we write *d*(*x*_*i*_, *v*_*i*_) for the minimal Hamming distance from a model of *v*_*i*_ to a model of *x*_*i*_.

**Theorem 4.2** *Let* $\bar{K}=K_{1},K_{2}\cdots K_{n}$ *and let* $\bar{\psi }=\psi_{1},\psi_{2}\cdots \psi_{n}$ *be sequences of formulas. If Tight_search* $\left(\bar{K},\bar{\psi }\right)$ *accepts then there exists ϕ such that K*_*i*_ *_d_ *ϕ* ⊨ *ψ*_*i*_ *for each i*.

**Proof**. Because for all *i* ∈ {1, 2⋯*n*}, *K*_*i*_ is consitent, so there exists *x*_*i*_ ⊨ *K*_*i*_ and $K_{i}=K_{i}^{\prime }\cup K_{i}^{\prime \prime }$, so $x_{i}⊧K_{i}^{\prime }$. Since the Algorithm 3 has been accepted, then $\forall w⊧K_{i}^{\prime },d\left(w,v_{i}\right)=d_{i}$, so we can conclude that *d*(*x*_*i*_, *v*_*i*_) = *d*_*i*_, thus there exists *ϕ* such that *K*_*i*_ *_d_ *ϕ* ⊨ *ψ*_*i*_ for each *i*.

Although the Flaccid_search Algorithm and the Tight_search Algorithm can obtain the desierd solution, we still need to further explore the complexity of the *LKG*(*_d_) problem. This problem can be seen as two stages: the stage one is the estimation of *v*_*i*_; the stage two is to check the the minimal Hamming distance between *v*_*i*_ and $K_{i}^{\prime }$.

**Theorem 4.3** *LKG*(*_d_) *is* $\sum_{2}^{P}$

**Proof**. In the initial estimation of *v*_*i*_, since the possibility of the *v*_*i*_ value is exponential, it can be verified by the non-deterministic Turing machine, so this problem is a *NP* problem.

After the initial estimation, we need to judge if $d\left(K_{i}^{\prime },v_{i}\right)<d_{i}$, but recall that $d(K_{i}^{\prime },v_{i})$ represents the the mininum Hamming distance here, so this check can be performed as follows. Guess a set of atomic propositional variables of size less than *d*_*i*_, and let $v_{i}^{\prime }$ be the interpretation obtained from *v*_*i*_ by switching the truth values of these variables. If $v_{i}^{\prime }⊧K_{i}^{\prime }$, then the minimum distance between $K_{i}^{\prime }$ and *v*_*i*_ is less than *d*_*i*_, since we need to obtain the minimal Hamming distance, so we must consider all possible value of $v_{i}^{\prime }$, this process is a *co* − *NP* problem.

Hence the entire *LKP*(*_d_) problem is the summary of a *NP* problem and a *co* − *NP* problem, so the complexity of which is $\sum_{2}^{P}$.

Recall that the complexity of the *PAP*(*_d_) problem is $\sum_{2}^{P}$ [15]. Compared with the *PAP*(*_d_) problem, the *LKG*(*_d_) problem is more complex (i.e. the initial knowledge set of agents is unkonwn) and has more application value, but through our algorithm, the *LKG*(*_d_) problem can be efficiently solved, which is the desired result.

## 5 Example

In this section, we are interested in a different question: can we use announcement finding to implement a feasible solution to a problem of practical interest? To answer this question, we describe a practical tool that uses announcement finding as the basis for a simulated robot controller.

Alice believes that eggs should be eaten every day, and milk cannot be drunk with yogurt, but people don’t know that Alice thinks that they should drink milk when they eat eggs. Bob believes that yogurt should be drunk and egg should not be eaten. We want to find an announcement that makes Alice believe in milk can be drunk with yogurt, and Bob believes in egg can be eaten with yogurt, and change their original knowledges as little as possible. For the above environment, we can build a model that the knowledge set of Alice is *K*_*Alice*_ and the knowledge set of Bob is *K*_*Bob*_, then the initial knowledge states defined as follows:

$$\begin{array}{c} \begin{array}{cc} & K_{Alice}^{\prime }=\left\{egg;milk\to \neg yogurt;\neg milk\to yogurt\right\}\\ & K_{Alice}^{\prime \prime }=\left\{egg\to milk\right\}\\ & K_{Bob}^{\prime }=\left\{\neg egg;yogurt\right\}\\ & K_{Bob}^{\prime \prime }=⌀ \end{array} \end{array}$$

After receive the announcement, the knowledge of Alice become that milk can be drunk with yogurt and knowledge of Bob become that egg can be eaten with yogurt. Therefore *ψ*_*Alice*_ = {*milk* ∧ *yogurt*} and *ψ*_*Bob*_ = {*yogurt* ∧ *egg*}. We want to find a formula *ϕ* such that:

$$\begin{array}{c} \begin{array}{cc} & K_{Alice}*\phi ⊢\psi_{Alice}\\ & K_{Bob}*\phi ⊢\psi_{Bob} \end{array} \end{array}$$

The input of the algorithm is $K_{Alice}^{\prime }$ and $K_{Bob}^{\prime }$, by executing the Algorithm 3, if we get *v*_1_ = ¬*egg* ∧ ¬*milk* ∧ *yogurt*, *v*_2_ = ¬*egg* ∧ *yogurt* and *d*_1_ = 1, *d*_2_ = 0, then according to the line 5 of the Algorithm 3, $\exists w⊧K_{Alice}^{\prime }$, *d*(*w*, *v*_1_) ≠ *d*_1_, so this solution is rejected. If we get *v*_1_ = *egg* ∧ ¬*milk* ∧ ¬*yogurt*, *v*_2_ = *egg* ∧ ¬*yogurt* and *d*_1_ = 1, *d*_2_ = 2, then according to the line 6 of the Algorithm 3, *v*_1_ ⊭ *ψ*_1_ and *v*_2_ ⊭ *ψ*_2_, so this solution is rejected. If we get *v*_1_ = *egg* ∧ *milk* ∧ *yogurt*, *v*_2_ = *egg* ∧ *yogurt* and *d*_1_ = 1, *d*_2_ = 1, then according to the line 5 of the Algorithm 3, $\forall w⊧K_{Alice}^{\prime },d\left(w,v_{1}\right)=d_{1}$ and $\forall w⊧K_{Bob}^{\prime },d\left(w,v_{2}\right)=d_{2}$, and according to the line 6 of the Algorithm 3, *v*_1_ ⊨ *ψ*_*Alice*_ and *v*_2_ ⊨ *ψ*_*Bob*_, thus *ϕ* = *v*_1_ ∧ *v*_2_, so this solution is accepted.

## 6 Experiment

In this section, we will validate the previously proposed Flaccid_search algorithm and Tight_search algorithm. Firstly, we need to provide the source of the dataset and the evaluation metrics. Then, we will design a series of experiments to verify their effectiveness and performance by comparing their performance on different datasets. Finally, we will analyze the experimental results, discuss the advantages and disadvantages of these two algorithms, and explore possible directions for improvement. Through these validations and analyses, we can better understand the characteristics of these two algorithms and provide guidance and reference for their practical applications.

### 6.1 Datasets

In this experiment, we utilized a publicly available dataset of Bitcoin OTC trust weighted signed network as the basis for our research, which cotain 6000 users and more than 35000 records. This dataset contains a wealth of information regarding who-trusts-whom network of people, which is pivotal to our investigation into Flaccid_search algorithm and Tight_search algorithm.

### 6.2 Implementation details

From this dataset, we extracted the score of trust and conducted data preprocessing and cleansing using Binary conversion. Subsequently, we employed Flaccid_search algorithm and Tight_search algorithm for data analysis and modeling, yielding several intriguing findings. All experiments were implemented using Python 3.9 and executed on an Apple M1 processor.

### 6.3 Comparison and evaluation

In the context of knowledge graph revision, our algorithm evaluation focuses on the existence rate (*ER*) and applicability rate (*AR*) of solutions. Specifically, we propose the Flaccid_search algorithm, which primarily focuses on *ER*, and the Tight_search algorithm, which primarily focuses on *AR*. This explicit classification aids in a deeper understanding of the performance characteristics of different algorithms in knowledge graph revision.

Based on the analysis of Table 1, it can be observed that as the parameter *d* varies, the Flaccid_search algorithm we proposed consistently outperforms the Exist_Ann algorithm in terms of the *ER* metric. This indicates that the Flaccid_search algorithm has a higher probability of finding solutions, demonstrating its superior practicality. Moreover, a more detailed examination reveals that the Exist_Ann algorithm exhibits a basically stable trend in *ER* values as *d* increases, whereas the Exist_Ann algorithm shows relatively lower and more fluctuating *ER* values. This trend suggests that the Flaccid_search algorithm is more reliable in finding solutions under different parameter settings, whereas the Exist_Ann algorithm’s solution capability is relatively unstable. Furthermore, a closer analysis reveals that the Exist_Ann algorithm maintains high *ER* values even at higher values of *d*, indicating its robustness and stability in finding solutions, even under complex conditions. In contrast, the Exist_Ann algorithm performs poorly at higher values of *d*, with significantly lower *ER* values, suggesting potential difficulties in finding solutions under complex conditions. In summary, the Flaccid_search algorithm we proposed is likely to have a higher probability of finding solutions in practical applications, demonstrating greater practicality and reliability, particularly when faced with complex conditions and parameter settings.

**Table 1:** Comparison of *ER*.

*d* = 0		*d* = 1		*d* = 2		*d* = 3	
Flaccid_search	Exist_Ann	Flaccid_search	Exist_Ann	Flaccid_search	Exist_Ann	Flaccid_search	Exist_Ann
0.2053	0.2053	0.9322	0.8720	0.4973	0.3285	0.2298	0.2102
0.8372	0.7268	0.448	0.288	0.5483	0.3553	0.3103	0.2458
0.5072	0.3258	0.4427	0.2948	0.8652	0.7563	0.3302	0.2390
0.3843	0.2543	0.9167	0.8522	0.3983	0.2795	0.3085	0.2300
0.2900	0.2215	0.3382	0.2415	0.9235	0.8607	0.4405	0.2923
0.3232	0.2367	0.8580	0.7478	0.3978	0.2615	0.5190	0.3343
0.2357	0.2102	0.5377	0.3468	0.4880	0.3213	0.8455	0.7353
0.2298	0.2102	0.4502	0.2905	0.9255	0.8635	0.2995	0.2433
0.2348	0.2138	0.2995	0.2433	0.9255	0.8635	0.4502	0.2905
0.2195	0.2077	0.8455	0.7353	0.4880	0.3213	0.5377	0.3468
0.2905	0.2410	0.5190	0.3343	0.3978	0.2615	0.8580	0.7478
0.2053	0.2053	0.4405	0.2923	0.9235	0.8607	0.3382	0.2415
0.2053	0.2053	0.3085	0.2300	0.3983	0.2795	0.9167	0.8522
0.2053	0.2053	0.3302	0.2390	0.8652	0.7563	0.4427	0.2948
0.2053	0.2053	0.3103	0.2458	0.5483	0.3553	0.4480	0.2880
0.2053	0.2053	0.2298	0.2102	0.4973	0.3285	0.9322	0.8720

**Fig 1:**
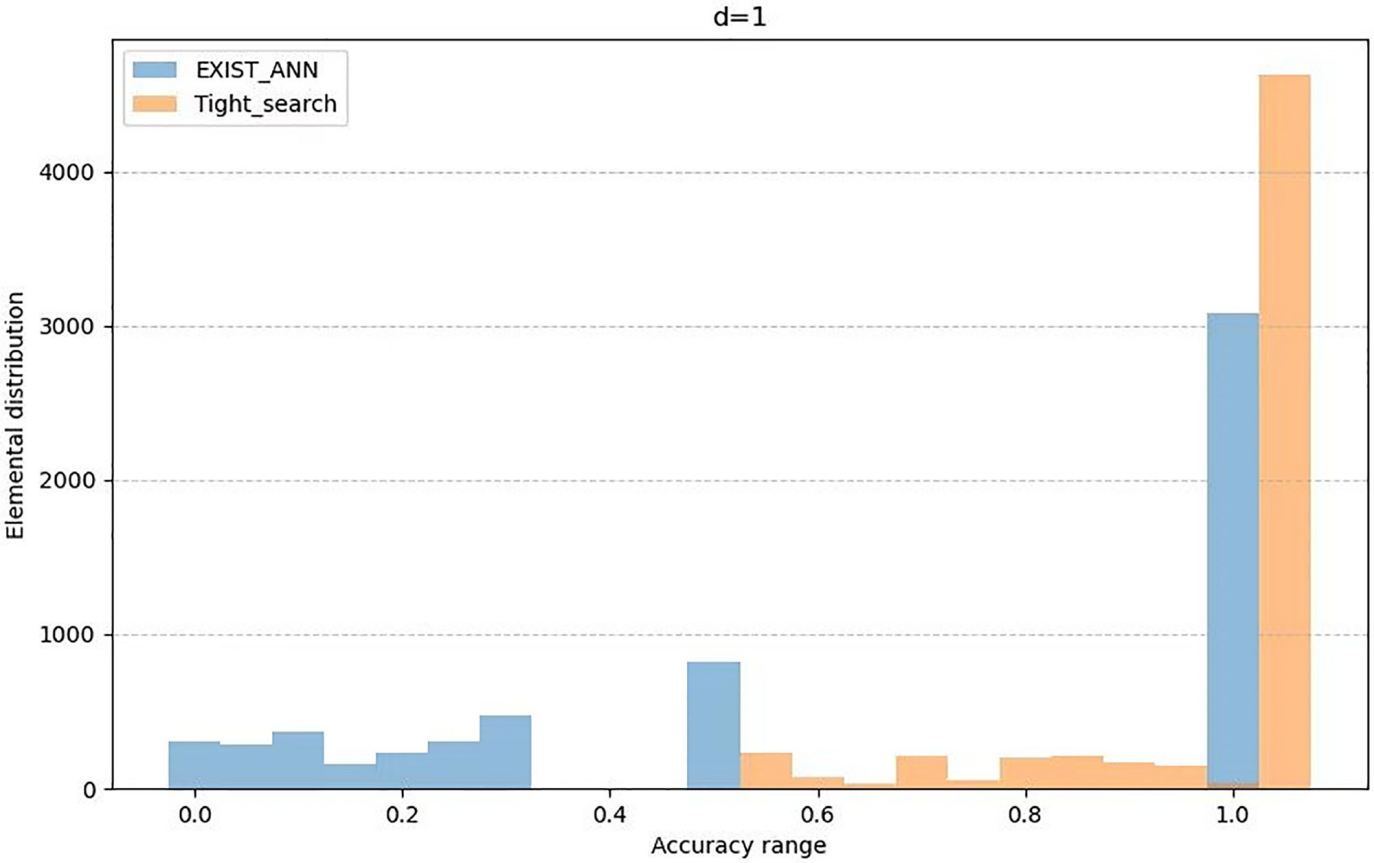
Element distribution when *d* = 1.

**Fig 2:**
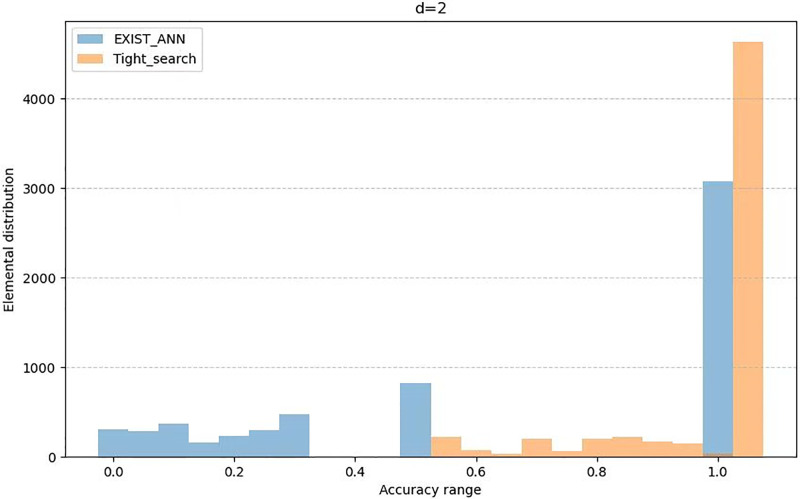
Element distribution when *d* = 2.

**Fig 3:**
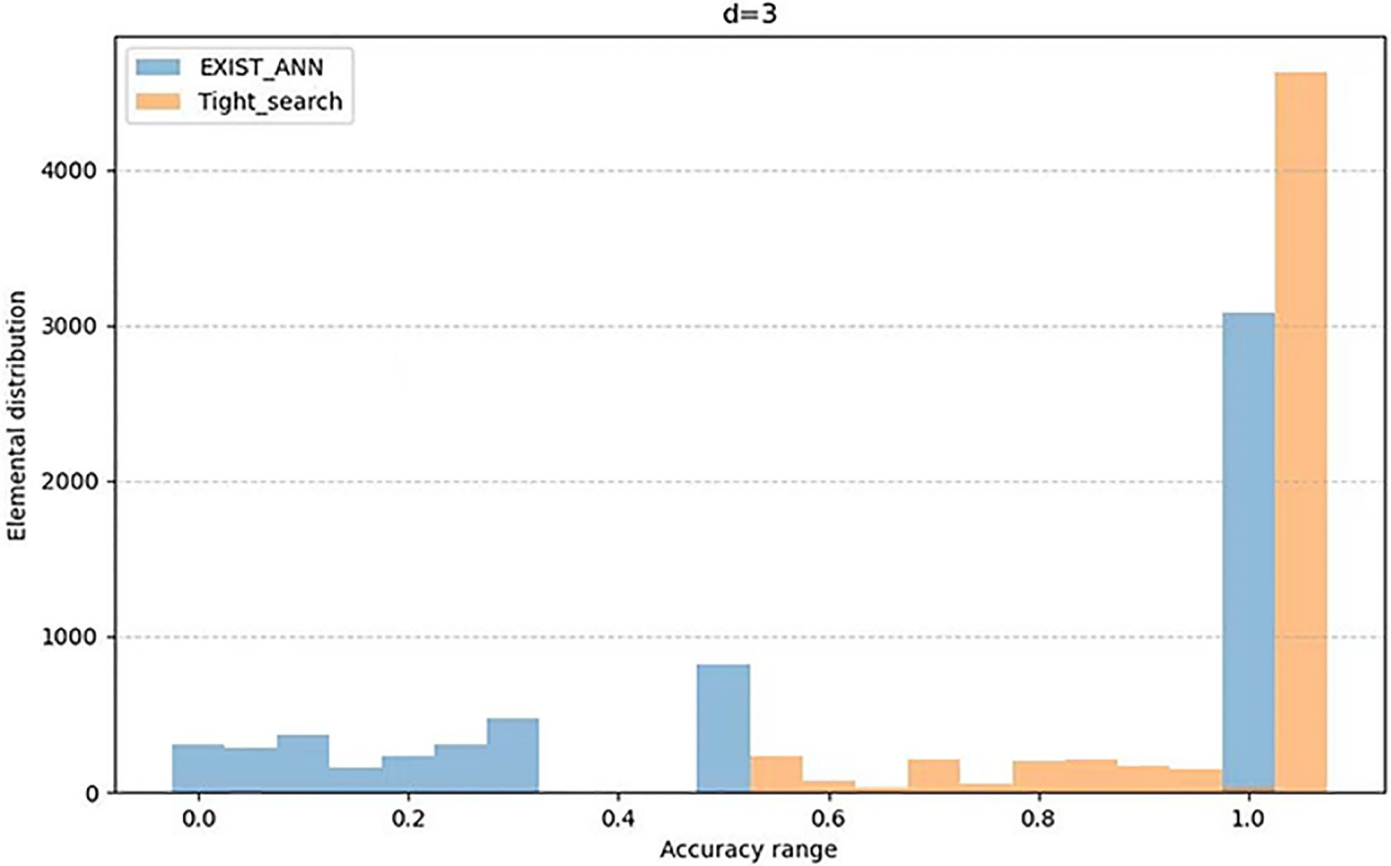
Element distribution when *d* = 3.

The Table 2 presents the average value of *AR* as variable *d* changes, while the Figs 1 to 3 illustrates the distribution of *AR* across different users as variable *d* changes. From the Table 2 and the Graph 1 to Graph 3, we can see that the Tight_search algorithm we propoed is clearly more widely distributed than the Exist_Ann algorithm in the high-accuracy interval. Moreover, it remains stable with the change of *d*. This indicates that the Tight_search algorithm exhibits better stability and consistency under these conditions and demonstrates a higher robustness to parameter *d* variations. Therefore, based on these observations, we can tentatively infer that the Tight_search algorithm may perform better on this problem.

**Table 2:** Comparison of *AR*.

Tight_search			Exist_Ann
*d* = 1	*d* = 2	*d* = 3	
0.9390	0.9379	0.9331	0.6443

## 7 Conclusion and future work

We have considered knowledge graph revision problem, in the context of the initial knowledge is unknown. In this setting, the past work can not come up with effective solutions, so we are conducting research on the Knowledge Graph Revision Problem with Limited Knowledge (LKG) problem and establishing the LKG problem model. To address this problem, we have proposed two approximate search algorithms: Flaccid_search and Tight_search, and analyzed the performance and characteristics of these two algorithms, we have proved that both the Flaccid_search Algorithm and the Tight_search.Algorithm can obtain the desired result. Finally, we explain the algorithm through an example and prove the effectiveness of the algorithm in solving knowledge graph revision problem.

In the future, we will look into more complicated situations, like modal logical settings or dynamic cognitive logical settings. We’ll work on making sure the entities and connections in knowledge graphs are more accurate and consistent. This means we’ll improve methods like connecting entities and extracting relationships. Also, we’ll concentrate on upgrading applications, especially in fields like healthcare and finance.
